# Dot-Size Dependent Excitons in Droplet-Etched Cone-Shell GaAs Quantum Dots

**DOI:** 10.3390/nano12172981

**Published:** 2022-08-28

**Authors:** Christian Heyn, Andreas Gräfenstein, Geoffrey Pirard, Leonardo Ranasinghe, Kristian Deneke, Ahmed Alshaikh, Gabriel Bester, Wolfgang Hansen

**Affiliations:** 1 Center for Hybrid Nanostructures (CHyN), University of Hamburg, Luruper Chaussee 149, 22761 Hamburg, Germany; 2 Physical Chemistry and Physics Departments, University of Hamburg, HARBOR Build., Luruper Chaussee 149, 22761 Hamburg, Germany; 3 The Hamburg Centre for Ultrafast Imaging, University of Hamburg, Luruper Chaussee 149, 22761 Hamburg, Germany

**Keywords:** quantum dot, droplet etching, photoluminescence, exciton, biexciton, lifetime, quantum efficiency, pseudopotential calculation

## Abstract

Strain-free GaAs quantum dots (QDs) are fabricated by filling droplet-etched nanoholes in AlGaAs. Using a template of nominally identical nanoholes, the QD size is precisely controlled by the thickness of the GaAs filling layer. Atomic force microscopy indicates that the QDs have a cone-shell shape. From single-dot photoluminescence measurements, values of the exciton emission energy (1.58...1.82 eV), the exciton–biexciton splitting (1.8...2.5 meV), the exciton radiative lifetime of bright (0.37...0.58 ns) and dark (3.2...6.7 ns) states, the quantum efficiency (0.89...0.92), and the oscillator strength (11.2...17.1) are determined as a function of the dot size. The experimental data are interpreted by comparison with an atomistic model.

## 1. Introduction

Semiconductor quantum dots (QDs) are essential building blocks for quantum information technology and future quantum networks. In this field, single QDs provide important functionalities as deterministic emitters for single photons [1] and entangled photon pairs [2,3,4,5] or an application as qubits [6]. In particular epitaxial QDs are advantageous due to their bright and stable optical emission [1]. Epitaxial QDs for optical applications are commonly fabricated using self-assembly mechanisms during molecular beam epitaxy (MBE). The self-assembly here provides a localization of the QD material, which is initially deposited as a planar layer. Two methods are often utilized for QD generation, both being based on energy minimization during crystal growth. In the Stranski–Krastanov (SK) mode, the strain energy caused by crystalline layers of different lattice constants drives the QD formation [7,8,9]. However, such SK-QDs are substantially strained, which causes piezoelectric fields that influence the optical properties. Strain-free QDs can be created using droplet-based techniques [10], where self-assembled metal droplets are generated in the Volmer–Weber growth mode. Here, the driving force for self-assembly is the minimization of surface and interface energies. After droplet formation, the droplets can be either re-crystallized to form, for instance, GaAs QDs during droplet epitaxy [11,12,13] or used to drill nanoholes during local droplet etching (LDE) [14,15,16]. The LDE nanoholes can be filled afterward with a material different from the substrate for QD generation [17,18,19].

The present study addresses LDE GaAs QDs that are formed by filling nanoholes etched with Al droplets in an AlGaAs substrate [20]. As an important point, the nanoholes are not completely filled for QD generation, and the QD size is precisely adjusted by the thickness of the GaAs layer deposited for hole filling. We focus here on the dot-size-dependent optical properties of the QDs, such as exciton emission, exciton-biexciton splitting, and lifetimes. The experimental data are interpreted by comparison with an advanced atomistic model.

## 2. QD Fabrication and Shape

**Figure 1:**
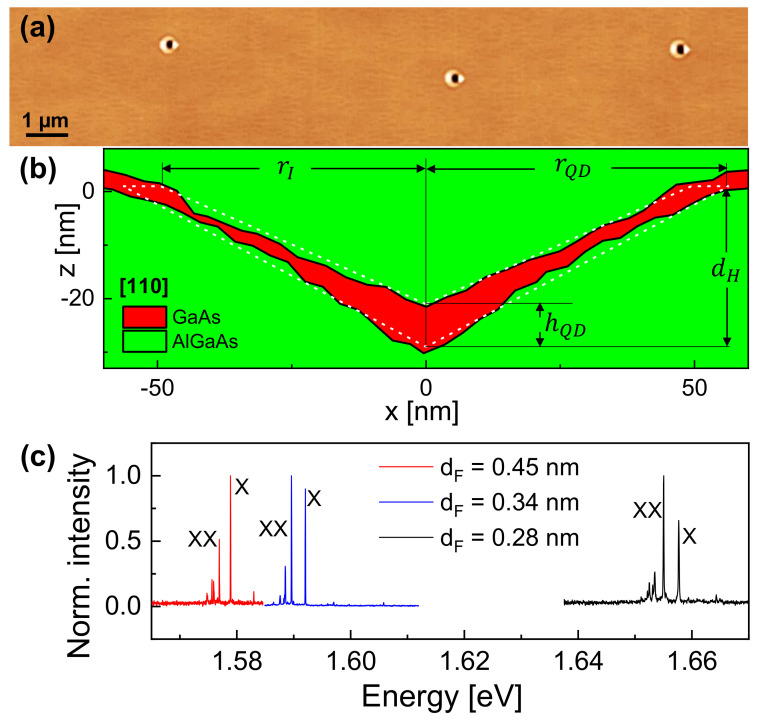
(**a**) Top-view atomic force microscopy (AFM) image of an AlGaAs surface after local droplet etching with Al. The low-density nanoholes are clearly visible. (**b**) AFM line scans along the [110] direction to illustrate the cross-section of a GaAs cone-shell QD (red) embedded in an AlGaAs matrix (green). The thickness of the GaAs filling layer is $d_{F}$ = 0.45 nm. The dashed white lines indicate the approximated QD shape used for the simulations, and the four model parameters are indicated. (**c**) Typical PL spectra from single cone-shell GaAs QDs with size varied by nanohole filling with different $d_{F}$. Exciton X and biexciton XX peaks are indicated.

The investigated samples are fabricated using solid-source MBE. Details of the local droplet etching and nanohole filling procedure are described earlier [20]. In brief, AlGaAs with an Al content of 33% (30% for samples for time-dependent experiments) is deposited on a (001) GaAs substrate to provide the confining barrier material for the QDs. Now, the Arsenic flux is reduced by a factor of about 100 in comparison to the growth of the AlGaAs layer, and Al droplets are deposited in the Volmer–Weber mode at a temperature of *T* = 625 °C. During a post-growth annealing step of *t* = 180 s, the initial Al droplets are transformed into nanoholes surrounded by a crystalline AlAs wall [16]. The hole shape is approximated here as an inverted cone. However, we note that recent experiments indicate a more complex shape with faceted inner surfaces inside the nanoholes [21]. The above growth conditions yield nanoholes with a low density of $2\times 10^{7}$ cm${}^{-2}$ and an average depth of 30 nm. An example of an atomically flat AlGaAs surface with low-density LDE nanoholes is shown in Figure 1a.

In the next step, the LDE nanoholes are filled for QD generation by deposition of a GaAs layer with thickness $d_{F}$. Since the process parameters for nanohole creation are nominally equal for all samples investigated here, the shape, as well as the size of the nanohole template, is assumed to be identical. That means the QD size is controlled directly by the filling layer thickness $d_{F}$. Finally, the QDs are overgrown by 80 nm AlGaAs as a barrier material.

For a characterization of the shape and size of the nanohole template, we have performed atomic force microscopy (AFM) measurements of a reference sample with unfilled nanoholes. For this, 10 nanoholes on the reference sample are imaged, and the average hole depth $d_{H}$ = 30 ± 2.3 nm is determined with respect to the level of the planar surface. The average radius of the hole opening at the plane of the planar surface is $r_{H}$ = 56.5 ± 7.4 nm. In the next step, a second sample now with filled nanoholes ($d_{F}$ = 0.45 nm) is fabricated, and again, the shape of 10 holes is imaged with AFM. The average hole depth after filling is 21.5 ± 1.9 nm. For an illustration of the general shape of the present QDs, we have selected an unfilled and a filled nanohole with respective depths close to the average values and created line scans along the [110] direction. To consider the thickness of the planar layer deposited for nanohole filling, the line scan of the filled nanohole is vertically shifted by $d_{F}$. Figure 1b shows the resulting line scans, where the difference between the filled and the unfilled nanohole is taken as QD. We note that the AFM data do not allow the determination of the exact QD size, since the line scans are taken from different samples. In particular, the level of the line scan of the filled QD and, thus, the estimated QD height are not reliable. Nevertheless, an inspection of Figure 1b suggests that the material of the GaAs QD probably completely covers the etched surface inside the LDE nanohole. Furthermore, the top part of the QD is not flat but shows an indentation caused by capillarity. Regarding this shape, we call these QDs cone-shell QDs [22].

For the simulations of the QD optical properties, an approximated rotational-symmetric shape is used that considers four parameters (Figure 1b): the cone-like nanohole-related parameters hole depth $d_{H}$ = 30 nm and hole radius $r_{H}$ = 56.5 nm, the radius $r_{I}$ = 49.4 nm of the of cone-like indentation and, finally, the QD height $h_{QD}$. We assume, in the following, that the nanohole-related parameters $d_{H}$, $r_{H}$ and the indentation radius $r_{I}$ are constant and, thus, are determined from the AFM data. The only free parameter is the QD height $h_{QD}$, which is controlled by the experimental filling layer thickness $d_{F}$.

## 3. Simulation Model

For the interpretation of optical data from the cone-shell GaAs QDs, we compare them with simulation results obtained using an advanced atomistic model. In the model, the cone-shell QDs are placed at the center of a periodic 210 × 210 × 70 $a_{0}^{3}$ supercell filled beforehand with the AlGaAs barrier material of lattice parameter $a_{0}$. The system is allowed to relax via a generalized valence force field (GVFF) model [23,24,25] that minimizes the strain energy. Then, the Pauli–Schrödinger equation is solved using screened empirical pseudopotentials [26] and the single-particle wave functions are decomposed into a strained linear combination of bulk bands (SLCBB) [27] on a 10×10×10*k-points* grid centered around the Γ point. The solution yields the electron (hole) wave functions $\phi_{e}$ ($\phi_{h}$) and their corresponding energies $\epsilon_{e}$ ($\epsilon_{h}$). Many-body exchange and correlations effects are included in the calculation through the use of screened configuration interaction (CI) [28,29,30], where the screening is performed with the Resta model [31]. The CI wave function is expanded onto a basis of excited Slater determinants restricted to the first 12 electron and 12 hole levels (including Kramers spin). The many-body Hamiltonian is then diagonalized, and in the case of the exciton, the matrix elements at play are given by $H_{he,h^{\prime }e^{\prime }}=\left(\epsilon_{e}-\epsilon_{h}\right)\delta_{hh^{\prime }}\delta_{ee^{\prime }}-J_{he,h^{\prime }e^{\prime }}+K_{he,h^{\prime }e^{\prime }}$ [28,29], where *J* and *K* are, respectively, the electron-hole Coulomb and exchange integrals, while δ is the Kronecker delta. The exciton energies $E_{X_{\nu }}$ are directly obtained while the characteristic lifetimes $\tau (X_{\nu })$ for each excitonic transition $X_{\nu }$ are calculated according to Fermi’s golden rule, using the full atomistic and correlated many-body wave function $|\Psi \left(X_{\nu }\right)\rangle$ as:

(1) $$\frac{1}{\tau (X_{\nu })}=\frac{e^{2}n}{3\pi \epsilon_{0}m_{0}^{2}c_{0}^{3}\hbar^{2}}E_{X_{\nu }}\sum_{e=e_{x},e_{y},e_{z}}|\langle \Psi \left(X_{\nu }\right)|e\cdot \hat{p}{\left|0\rangle \right|}^{2},$$

where |0〉 is the Fermi vacuum state and $X_{\nu }$ with ν∈{0,1,2,3} representing a substrate of the exciton manifold, usually consisting of two dark states and two bright states. e is a polarization unit vector while $\hat{p}$ is the momentum operator. Furthermore, *e* is the elementary charge, *n* the refractive index of the AlGaAs barrier, $\epsilon_{0}$ the vacuum permittivity, $m_{0}$ the electron mass, and $c_{0}$ the speed of light.

## 4. QD-Size Dependent Exciton Energies

The optical emission from single GaAs cone-shell QDs is studied using a low-temperature micro-photoluminescence (PL) setup. The samples are located inside an optical cryostat at a temperature of *T* = 4 K and excited either by a CW green laser (λ = 532 nm) or a pulsed red laser (λ = 638 nm, pulse duration < 90 ps) for the lifetime experiments. The emission is analyzed by a *f* = 750 mm monochromator in combination with a liquid-nitrogen cooled CCD camera, which is replaced by an avalanche photodiode for lifetime measurements.

Figure 1c shows typical PL spectra from single QDs, where the respective size $h_{QD}$ is varied by the thickness $d_{F}$ of the GaAs layer deposited for nanohole filling. Clearly visible are the exciton X and biexciton XX peaks. The identification is performed using excitation power-dependent measurements as described in an earlier paper [32]. The spectra demonstrate that the exciton and biexciton peak energies $E_{X}$, $E_{XX}$ can be precisely adjusted by the value of $d_{F}$.

**Figure 2:**
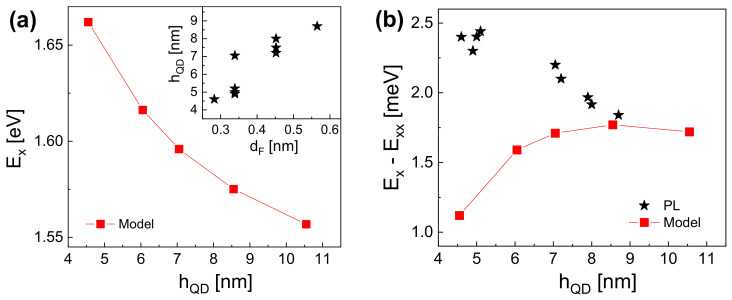
(**a**) Relation between the exciton energy $E_{X}$ and the quantum dot height $h_{QD}$ computed with the atomistic model. The inset shows pairs of $h_{QD}$ and $d_{F}$, where the values of $E_{X}$ computed with the model agree with the PL measurements. (**b**) Exciton–biexciton splitting measured with PL together with model results.

Nevertheless, the relation between the experimentally controlled value of $d_{F}$ and the QD size $h_{QD}$ is not known so far. As is described above, the AFM data are not accurate enough for a precise determination. Therefore, we estimate the relation $h_{QD}\left(d_{F}\right)$ from a comparison with simulation results (Figure 2a). For this, we measure the exciton energy $E_{X}$ from single QDs on the four different samples, where the filling layer thickness $d_{F}$ used for the respective sample fabrication gives the *x*-axis in the inset of Figure 2a. The height $h_{QD}$ of the individual QDs in the inset of Figure 2a is determined by comparison with the model results in Figure 2a using the measured $E_{X}$. This relation is used in the following for the determination of $h_{QD}$ from the measured exciton energies.

In Figure 2b, the exciton–biexciton splitting is plotted as a function of $h_{QD}$. The PL data show a decrease from 2.5 meV down to 1.8 meV with increasing QD size. The atomistic model results agree with the experiments for $h_{QD}>$ 8 nm. However, for smaller dots, the simulated trend is inverse to that of the PL results. A possible explanation for this discrepancy is a deviation of the QD shape for smaller dots from the assumed shape in Figure 1b.

## 5. QD Lifetimes

**Figure 3:**
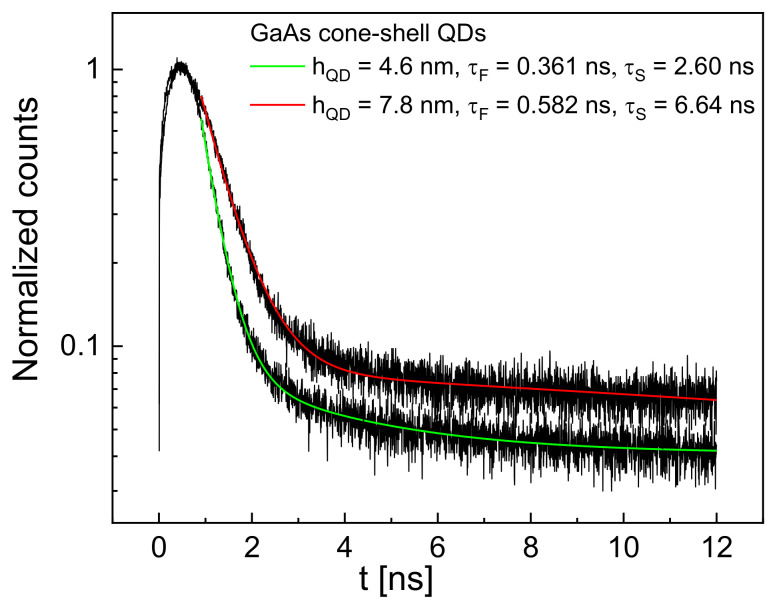
Examples of the normalized time-dependent exciton-peak PL intensity for samples with $h_{QD}$ = 4.6 nm ($d_{F}$ = 0.28 m) and $h_{QD}$ = 7.8 nm ($d_{F}$ = 0.45 nm). Fit results assuming a biexponential decay with a fast process $\tau_{F}$ and a slow process $\tau_{S}$ are also shown.

In the next step, we performed time-dependent PL experiments. Here, four samples are studied with GaAs cone-shell QDs of varied size: one sample with $d_{F}$ = 0.28 nm, one with $d_{F}$ = 0.34 nm, and two samples with $d_{F}$ = 0.45 nm. The corresponding values of $h_{QD}$ are determined via a comparison with the simulated $E_{X}$, as described above. In Figure 3, examples of the time-dependent exciton PL intensity are plotted. After an initial fast increase of about 0.45 ns related to the filling of the QD with excitons, afterward, the intensity decreases due to recombination events. Since fitting with a simple exponential decay yields only a poor reproduction of the experimental data, we use a biexponential decay, which provides a much better agreement. This is in accordance with previous observations [33]. A biexponential fit of the PL intensity

(2) $$I\left(t\right)=A_{F}\exp\left(-t/\tau_{F}\right)+A_{S}\exp\left(-t/\tau_{S}\right)+I_{0}$$

provides five parameters: $A_{F}$, $\tau_{F}$, $A_{S}$, $\tau_{S}$, and $I_{0}$. Figure 3 shows examples where a fast decay with $\tau_{F}$ and a slow decay $\tau_{S}$ can be clearly identified.

The fitted parameters are analyzed following Narvaez et al. [34] to obtain the characteristic QD lifetimes. An exciton can be interpreted as a five-level system with two excited fine-structure split bright states |b〉, $|b^{\prime }\rangle$, two excited fine-structure split dark states |d〉, $|d^{\prime }\rangle$, and the ground state |0〉. For bright state excitons, the spin configuration allows high transition rates $R_{b0}$ from |b〉 to |0〉 and $R_{b^{\prime }0}$ from $|b^{\prime }\rangle$ to |0〉, whereas the transition rates $R_{d0}$, $R_{d^{\prime }0}$ for dark states are much smaller. Since the atomistic model indicates $R_{b0}≃R_{b^{\prime }0}$, we average the bright state transitions, and the rate of radiative recombinations of the bright states becomes $R_{B}=\left(R_{b0}+R_{b^{\prime }0}\right)/2$. On the other hand, since the model results indicate $R_{d0}\gg R_{d^{\prime }0}$, we use $R_{D}=R_{d0}$ for the rate of radiative recombinations of the dark states. This approach simplifies the model into a three-level system with bright |B〉, dark |D〉, and ground state |0〉. The time-dependent population probability $n_{B}\left(t\right)$ of the bright state is reduced by $R_{B}$ and the dark state population $n_{D}\left(t\right)$ by $R_{D}$. In addition, both populations are modified by spin-flip events with rate $R_{BD}$ from bright to dark and $R_{DB}$ vice versa. This approach is summarized in the following rate equation

(3) $$\left(\begin{array}{c} {\dot{n}}_{B}\left(t\right)\\ {\dot{n}}_{D}\left(t\right) \end{array}\right)=\left(\begin{array}{cc} -R_{B}-R_{BD} & R_{DB}\\ R_{BD} & -R_{D}-R_{DB} \end{array}\right)\left(\begin{array}{c} n_{B}\left(t\right)\\ n_{D}\left(t\right) \end{array}\right)$$

Narvaez et al. assume $R_{BD}=R_{DB}=2R_{SF}$, where the factor of two before the spin-flip rate $R_{SF}$ considers the above splitting of the states by the fine structure. With the approximation $n_{D}\left(0\right)=n_{B}\left(0\right)=A_{F}+A_{S}$, the radiative and nonradiative recombination rates can be extracted from a biexponential fit (Equation (2)) of the PL intensity decay by

(4) $$R_{B}=+\frac{A_{F}}{A_{F}+A_{S}}\tau_{F}^{-1}+\frac{A_{S}}{A_{F}+A_{S}}\tau_{S}^{-1}$$

and

(5) $$R_{D}=-\frac{A_{S}}{A_{F}-A_{S}}\tau_{F}^{-1}+\frac{A_{F}}{A_{F}-A_{S}}\tau_{S}^{-1}$$

**Figure 4:**
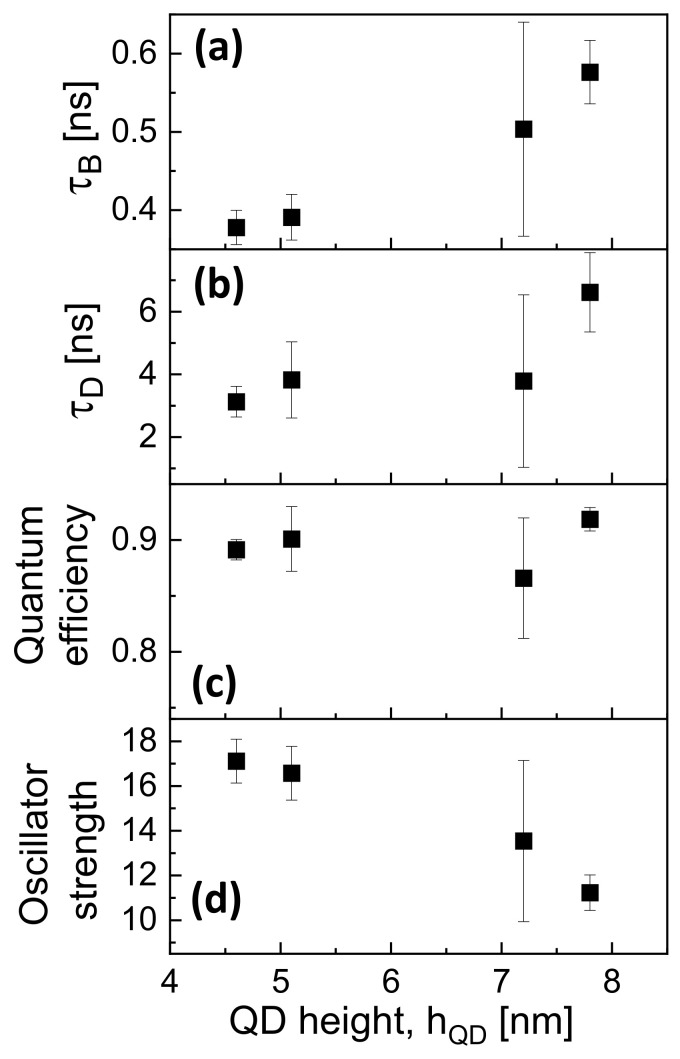
Dot-size dependent QD optical parameters extracted from biexponential fits of the time-dependent exciton peak intensity using the approach of Narvaez et al. [34]. Radiative lifetimes of (**a**) bright and (**b**) dark states, (**c**) quantum efficiency, and (**d**) oscillator strength.

Fits from four to five individual QDs on each of the above samples are analyzed, and the radiative recombination rate is determined using Equation (4). The corresponding radiative lifetimes $\tau_{B}=1/R_{B}$ are plotted in Figure 4a as a function of the QD size $h_{QD}$. We find an increase in $\tau_{B}$ from 0.37 ns up to 0.58 ns with increasing $h_{QD}$. The radiative lifetime of the dark states $\tau_{D}=1/R_{D}$ determined using Equation (5) is roughly 10 times longer and increases with the dot size from 3.2 ns up to 6.7 ns (Figure 4b).

Furthermore, we calculate the quantum efficiency from $\eta =R_{B}/\left(R_{B}+R_{D}\right)$ and the oscillator strength from $f=6\pi \epsilon_{0}m_{0}c_{0}^{3}R_{B}/\left(e^{2}n\omega_{0}^{2}\right)$, with the frequency $\omega_{0}$ of light. The results are shown in Figure 4c,d. For η, a clear dependence on the QD size is not visible, and we determine for the four samples an average value of the quantum efficiency of η = 89.4%. In contrast to that, the average oscillator strength decreases with increasing QD size from *f* = 17.1 down to 11.2.

These results on cone-shell GaAs QDs are now compared with data obtained by Tighineanu et al. [33] from GaAs QDs fabricated using droplet epitaxy. There, three QDs are studied without a clear indication of the respective QD size. The radiative lifetimes range from $\tau_{B}$ = 0.68 to 0.79 ns, which is slightly above the present values, and the dark state ones from $\tau_{D}$ = 1.67 to 3.57 ns, which is roughly half of the present results. The difference in $\tau_{B}$ can be related to a different QD shape and size, whereas the shorter $\tau_{D}$ can be caused by a higher density of crystal defects. For the quantum efficiency η, values between 0.69 and 0.78 are given, and the oscillator strength *f* ranges from 8.2 to 9.4. Both values are below the present data. This lower optical brightness of the droplet epitaxial QDs is in accordance with the longer radiative and the shorter nonradiative lifetimes, where the latter can be attributed to defect formation during the low-temperature growth of the capping layer [33].

**Figure 5:**
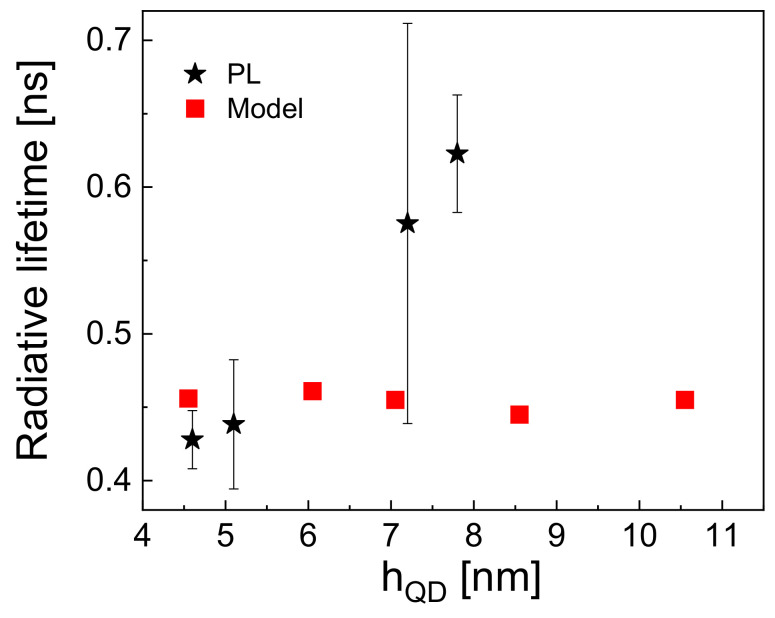
Experimental and simulated radiative lifetimes as a function of the quantum dot size.

Finally, Figure 5 demonstrates a reasonable agreement of experimental radiative lifetimes with simulation results obtained using the atomistic model. The experimental lifetimes increase from 0.37 ns up to 0.58 ns with increasing $h_{QD}$, whereas the model results only show a weak dependence on $h_{QD}$. The slight deviation might be related to a deviation of the QD shape from the shape assumed in the model.

## 6. Conclusions

The present study combines experimental data of the dot-size dependent optical properties of GaAs cone-shell QDs with simulation results using an advanced atomistic model. In particular, the energies of the ground-state exciton and biexciton, as well as bright and dark exciton lifetimes, are investigated. We find a clear influence of the QD size on the ground-state energy, lifetimes, and oscillator strength, whereas the exciton-biexciton splitting and quantum efficiency are almost size-independent. A disagreement between the experimental and simulated exciton-biexciton splitting for small QDs and slight deviations of the radiative lifetimes might indicate that the approximated QD shape used for the modeling should be improved. The present approach for the QD shape is taken from AFM images measured on different samples and allows only a qualitative estimation of QD shape and size. On the other hand, both the exciton–biexciton splitting as well as the lifetime depend on the detailed shape of the electron and hole wave functions inside the QD. The splitting $E_{X}-E_{XX}$ occurs via attractive and repulsive Coulomb interactions and correlation effects, and the lifetime is calculated via the wave-function overlap. Obviously, the present cone-shell QDs significantly deviate from a simple sphere, which indicates complex wave functions with a strong modification to the dot shape. As a further outcome of the present study, a comparison with data from droplet epitaxial GaAs QDs [33] demonstrates for the present LDE cone-shell GaAs QDs an about 16% higher quantum efficiency and about 45% higher oscillator strength, suggesting them as bright photon emitters for applications in quantum technology.

## Data Availability

Not applicable.
